# Supplementary material for: Carbon sequestration by multiple biological pump pathways in a coastal upwelling biome
Source: Nat Commun. 2023 Apr 11;14:2024. doi: 10.1038/s41467-023-37771-8 (PMC10090055; doi:10.1038/s41467-023-37771-8)
Supplement: Supplementary file 1 — Supplementary Information [file 41467_2023_37771_MOESM1_ESM.pdf]

Supplementary material for:

Carbon sequestration by multiple biological pump pathways in a coastal upwelling biome

Michael R. Stukel[1,2], John P. Irving[1,2], Thomas B. Kelly[1,3], Mark D. Ohman[4], Christian K. Fender[1], Natalie Yingling[1]

[1]Earth, Ocean, and Atmospheric Science Dept., Florida State University, Tallahassee, FL USA

[2]Center for Ocean Atmosphere Prediction Studies, Florida State University, Tallahassee, FL USA

[3]College of Fisheries and Ocean Sciences, University of Alaska Fairbanks, Fairbanks, AK USA

[4]Scripps Institution of Oceanography, University of California-San Diego, La Jolla, CA USA

This document contains supplementary tables S1 – S3 followed by supplementary methods.

SUPPLEMENTARY TABLES S1 – S3

Supplementary Table S1. Ecosystem parameters

Cycle	Surface Temperature (°C)	Surface Nitrate (µmol L^{-1})	Surface Chl (µg Chl *a* L^{-1})	Net Primary Productivity (mmol C m^{-2} d^{-1})
605.1	12.19	7.78	5.75	347.51
605.2	14.64	0.08	0.10	24.72
605.4	14.75	0.86	0.90	120.18
605.5	16.39	0.12	0.10	38.20
704.1	12.45	6.33	1.57	101.23
704.2	14.24	0.05	0.20	47.76
704.4	12.33	6.99	1.29	191.27
810.1	17.04	0.31	0.53	45.94
810.2	16.98	0.18	0.24	39.81
810.3	15.92	0.30	0.75	73.97
810.4	15.95	0.17	1.03	55.97
810.5	14.96	1.98	1.42	139.15
810.6	17.14	0.16	0.21	26.29
1106.1	14.63	2.14	0.49	58.52
1106.2	15.31	0.04	0.11	25.70
1106.3	14.30	5.60	0.53	82.13
1106.4	16.01	0.27	3.18	83.05
1106.5	16.04	0.01	0.13	38.28
1208.1	14.78	1.29	2.64	120.89
1208.2	15.42	0.45	0.41	83.43
1208.3	15.61	0.79	0.86	62.39
1208.4	17.05	0.03	0.09	16.46
1208.5	15.57	1.65	0.59	82.50

1408.1	16.63	0.04	0.56	32.20
1408.2	16.93	0.05	0.66	26.69
1408.3	18.69	0.04	0.19	23.31
1408.4	19.18	0.03	0.12	9.93
1408.5	19.83	0.02	0.08	10.99
1604.1	17.36	0.03	NaN	18.31
1604.2	15.21	0.07	0.12	21.76
1604.3	13.62	3.45	0.89	72.11
1604.4	14.42	1.13	4.11	138.17
1706.1	12.98	6.23	10.31	564.11
1706.2	12.91	7.74	3.17	242.11
1706.3	14.95	1.62	0.76	108.38
1706.4	15.08	2.90	0.25	45.29
1908.1	13.46	NaN	4.60	1403.95
1908.2	15.64	NaN	1.84	733.48
1908.3	16.67	NaN	0.44	367.10
1908.4	20.57	NaN	0.10	514.66
2107.2	15.56	NaN	2.01	NaN
2107.3	18.94	NaN	0.08	NaN

Supplementary Table S2 – Active transport.

	Lagrangian Cycle								
	**704.1**	**704.2**	**704.4**	**810.1**	**810.2**	**810.3**	**810.4**	**810.5**	**810.6**
**Depth (m)**	**Active Transport due to Respiration + Excretion (mmol C m^{-2} d^{-1})**								
**100**	2.495	0.622	5.966	3.829	1.757	3.262	1.500	6.026	0.775
**150**	2.154	0.657	5.343	3.854	1.748	3.231	1.403	3.354	0.694
**200**	1.969	0.624	5.456	3.748	1.530	2.957	1.181	2.064	0.705
**250**	1.706	0.568	1.488	3.104	1.451	2.429	0.916	1.596	0.727
**300**	1.871	0.516	1.481	2.856	1.342	2.161	0.873	1.609	0.756
**350**	1.920	0.331	1.475	2.684	1.230	2.015	0.824	1.493	0.670
**400**	1.809	0.278	1.280	2.398	0.683	1.689	0.678	NaN	NaN
**450**	1.569	0.177	0.847	1.616	0.503	1.000	0.423	1.027	0.256

Supplementary Table S3 - Spearman's rank correlation between the efficiency of an export pathway (row = export pathway / net primary productivity) and an ecosystem metric (column)

	Surface Temperature (°C)	Surface Chl (µg Chl *a* L^{-1})	Net Primary Productivity (mmol C m^{-2} d^{-1})	Surface Nitrate (µmol L^{-1})
**Sinking/NPP**	0.46 (p=0.0053)	-0.52 (p=0.0017)	-0.78 (p=4e-07)	-0.56 (p=0.00096)
**Active Transport/NPP**	0.47 (p=0.21)	-0.033 (p=0.95)	-0.17 (p=0.68)	0.28 (p=0.46)
**Subduction/NPP**	0.52 (p=0.071)	-0.77 (p=0.0029)	-0.87 (p=6.8e-05)	-0.76 (p=0.0036)

SUPPLEMENTARY METHODS

Quantifying carbon flux due to subduction and vertical mixing

The physical processes that transport organic carbon to depth (sometimes referred to as the large-scale physical pump, the mixed layer pump, and the eddy subduction pump) act across multiple spatial scales from the submesoscale to basin scale. Consequently, it is not possible to measure carbon transport resulting from all of these interrelated processes (which hereafter we refer to simply as "subduction") directly from field measurements. Hence, we quantified rates of particulate organic carbon subduction by combining a biogeochemical data assimilation approach, a physical circulation data assimilation approach, and a Lagrangian particle tracking model[1]. We outline each of those approaches here, but refer readers to ref[1] for additional details.

Biogeochemical data-assimilation submodule: The fundamental premise in our modeling approach is that 1) particles are simultaneously transported by ocean circulation and their own gravitational settling and that 2) gravitational settling rates should be modeled as a continuous spectrum of sinking speeds because a diverse suite of sinking particles with highly variable settling rates exist in the ocean. We made the *a priori* assumption that the spectrum of sinking particle speeds in the CCE would be best modeled by considering two classes of particles, both of which have sinking speed distributions described by a log-normal distribution (i.e., most particles sink slowly but some sink substantially faster) but with different mean sinking speeds for each particle class. These particle classes were chosen to be representative of phytoplankton (which mostly have very slow settling rates) and fecal pellets (which typically dominate sinking flux in the CCE[2]). We thus define the production rate of particles as a function of sinking speed (S) as:

$$PP(S) = \tau \frac{1-\Phi}{\sigma_1\sqrt{2\pi}S} \exp\left(-\frac{(ln(S)-\mu_1)^2}{2\sigma_1{}^2}\right) + \tau \frac{\Phi}{\sigma_2\sqrt{2\pi}S} \exp\left(-\frac{(ln(S)-\mu_2)^2}{2\sigma_2{}^2}\right) \quad \text{Eq. S1}$$

where τ is the total particle production rate, Φ is the ratio of fecal pellet production to total particle production, and μ_1 and μ_2 are parameters related to the median sinking rate of phytoplankton and fecal pellets, respectively. σ_1 and σ_2 define the variability in the sinking rates of phytoplankton and fecal pellets, respectively and we assume a priori that both of these parameters are equal to 1, because this generates realistic variability in particle sinking speeds. An additional parameter used in the subduction model was the particle remineralization rate (λ). Because there is a limit to the number of parameters that could be uniquely defined from the field data, we further chose to assume a fixed ratio of 100 between the median sinking speeds of phytoplankton and fecal pellets. We thus assume that fecal pellet settling rates are two orders of magnitude greater than those of phytoplankton, a difference in settling rates that was chosen based on multiple studies showing phytoplankton settling velocities on the order of 1 m d^{-1} and fecal pellet settling rates on the order of 100 m d^{-1} (e.g., [3-7]). Thus $\mu_2=\ln(100\times\exp(\mu_1))$. This left us with four unknown parameters that needed to be fitted from our field data: τ, Φ, μ_1, and λ.

To objectively define these four parameters, we used field data from Lagrangian experiments conducted during three process cruises of the CCE LTER program (see main text for descriptions of these sampling programs). During each of these 14 Lagrangian experiments (duration = 2 – 5 days), we quantified NPP using $H^{14}CO_3^-$ uptake daily at 6 – 8 depths spanning the euphotic zone[2], we measured POC concentrations at 8 depths in the euphotic zone, we determined sinking particle flux using sediment traps or ^{238}U-^{234}Th disequilibrium[8,9], and we quantified mesozooplankton grazing rates using the gut pigment method[10]. Our data assimilation process began by defining the flux of sinking particles as a function of speed (S) and depth (z):

$$Flux(z,S) = PP(S) \times e^{-\lambda(z-d_{prod})/S} \quad \text{Eq. S2}$$

where λ is again the remineralization rate (units of d^{-1}) and d_{prod} is the mean depth of particle production (which we calculated from our $H^{14}CO_3^-$ uptake profiles). We can then define the following relationships between our measurements and Equations S1 and S2:

$$^{14}CPP = \int_{S=S_{min}}^{S=S_{max}} PP(S)dS \quad \text{Eq. S3}$$

$$Export_z = \int_{S=S_{min}}^{S=S_{max}} Flux(z,S)dS \quad \text{Eq. S4}$$

$$d_{prod} = \frac{\int z \times {}^{14}CPP(z)dz}{\int {}^{14}CPP(z)dz} \quad \text{Eq. S5}$$

Because the flux of sinking particulate carbon (at a specific depth) with any particle sinking speed will simply be equal to the carbon content of particles with that sinking speed (at that same depth) times the sinking speed, we can define the carbon content of particles as a function of sinking speed and depth as C(z,S) and relate this to our measured vertically integrated POC concentrations in the water column as:

$$POC_{total} = \int_{z=d_{prod}}^{z=d_{POC}} \int_S C(z,S)dSdz \quad \text{Eq. S6}$$

where d_{POC} is the maximum depth at which POC measurements are made (if shallower than the depth of the export measurement).

We can further relate Φ (the ratio of fecal pellet production to total particle production) through the equation:

$$\Phi = \frac{Graz \times EE}{\int {}^{14}CPP(z)dz} \quad \text{Eq. S7}$$

where Graz is the measured mesozooplankton grazing rate (units of mg C m^{-2} d^{-1}) and EE is the egestion efficiency of zooplankton which was assumed to be 0.3 following ref[11].

This leaves us with 5 equations (S3 – S7) that can be used to define 5 parameters (τ, Φ, μ_1, λ, and d_{prod}). To solve this system of equations we used a grid search approach while assuming that sinking speeds varied from 1 mm d^{-1} to 1 km d^{-1}. The resultant parameters used for the particle subduction model are given in Table S4:

Table S4: Parameters for the particle model as determined from field data collected on CCE LTER process cruises.

Cycle	τ (mg C m^{-2} d^{-1})	λ (d^{-1})	exp(μ_1) (m d^{-1})	exp(μ_2) (m d^{-1})	Φ (unitless)
0605-1	4170	0.29	1.44	143.8	0.10
0605-2	539	0.10	0.95	95.1	0.11
0605-4	1442	0.21	2.80	280.1	0.06
0605-5	458	0.07	1.43	143.3	0.05
0704-1	1215	0.18	0.09	9.3	0.49
0704-2	573	0.10	0.15	14.9	0.10
0704-4	2295	0.33	0.14	14.3	0.23
0810-1	551	0.08	1.68	167.6	0.03
0810-2	478	0.10	1.64	164.3	0.08
0810-3	888	0.15	0.63	62.5	0.11
0810-4	672	0.04	1.72	171.6	0.05
0810-5	1670	0.27	0.22	22.2	0.21
0810-6	316	0.05	2.12	211.7	0.17

Data-assimilating circulation model: We used physical circulation state estimates derived using the ROMS 4DVAR (Regional Ocean Modeling System, 4-Dimensional VARiational data-assimilation system[12]) physical circulation model. The model configuration had 9-km horizontal resolution with 42 terrain-following vertical layers (minimum thickness = 1-m near surface in coastal regions; maximum thickness = 300 m near bottom in open ocean). Thirty-day state estimates for the time period of our cruises were developed by sequentially updating model initial conditions and surface forcing to minimize a cost function relating model outputs to remotely sensed outputs as well as in situ physical measurements (temperature, salinity) from our cruises[13,14]. These state estimates were extended 30 days into the future (post-cruise and past the assimilated time frame) with a free-running model estimate, to ensure that dynamically consistent fields were available to force advection of simulated particles for 30 days following each Lagrangian experiment conducted in the field. State estimates were saved with 8-hourly temporal resolution.

Lagrangian particle simulations: To simulate the four-dimensional particle trajectories that result from a combination of sinking and physical circulation we used a modified version of the LTRANS Fortran package[15]. LTRANS advects particles using ROMS velocity and eddy diffusivity fields as well as “behavior”, which here refers to particle sinking speeds. The modified package allows each individual particle to have a sinking speed selected randomly from the particle sinking speed distributions defined in Eq. S1 using the parameters in the table above. For each of the 14 Lagrangian experiments conducted at sea, we released 10,000 simulated particles. Particles were released at random locations along the in situ Lagrangian experiment trajectories and at depths proportional to the measured vertical profiles of NPP. Particles were tracked for a period of 30 days (with one-hour time step) and remineralized according to the remineralization constants (λ) listed above. Importantly, all particles sank (but at very different speeds) and were transported by physical circulation. Particles were determined to have been “subducted” across a particular depth horizon if they were transported across that depth horizon during the physical time step in LTRANS (rather than the biological/sinking time step). Conversely, particles that were transported across that depth horizon during the biological/sinking time step were considered to have sunk across that depth horizon. Note that this explicitly allows particles to contribute to both of these BCP pathways at different depths, as they can be subducted across one depth horizon and then sink past another horizon or vice versa. Carbon flux associated with “subduction” was determined based on the carbon existing in the particle at the time of subduction. We note however that this “subduction” really refers to downward physical transport associated with subduction and vertical mixing generated by multiple physical mechanisms (e.g., it combines the “eddy-subduction pump”, “mixed-layer pump”, and “large-scale physical pump” as defined in ref[16]).

SUPPLEMENTARY REFERENCES

1 Stukel, M. R., Song, H., Goericke, R. & Miller, A. J. The role of subduction and gravitational sinking in particle export, carbon sequestration, and the remineralization length scale in the California Current Ecosystem. *Limnol. Oceanogr.* **63**, 363-383 (2018). https://doi.org:10.1002/lno.10636

2 Morrow, R. M. *et al.* Primary Productivity, Mesozooplankton Grazing, and the Biological Pump in the California Current Ecosystem: Variability and Response to El Niño. *Deep-Sea Res. I* **140**, 52-62 (2018). https://doi.org:10.1016/j.dsr.2018.07.012

3 Smayda, T. J. The suspension and sinking of phytoplankton in the sea. *Oceanography and Marine Biology Annual Review* **8**, 353-414 (1970).

4 Smayda, T. J. & Bienfang, P. K. Suspension properties of various phyletic groups of phytoplankton and tintinnids in an oligotrophic subtropical system. *Mar. Ecol.* **4**, 289-300 (1983).

5 Turner, J. T. Zooplankton fecal pellets, marine snow and sinking phytoplankton blooms. *Aquatic Microbial Ecology* **27**, 57-102 (2002).

6 Stukel, M. R., Mislan, K. A. S., Décima, M. & Hmelo, L. in *Eco-DAS IX Symposium Proceedings* (ed P. F. Kemp) 49-76 (Association for the Sciences of Limnology and Oceanography, 2014).

7 Small, L. F., Fowler, S. W. & Unlu, M. Y. Sinking rates of natural copepod fecal pellets. *Mar. Biol.* **51**, 233-241 (1979).

8 Stukel, M. R., Landry, M. R., Benitez-Nelson, C. R. & Goericke, R. Trophic cycling and carbon export relationships in the California Current Ecosystem. *Limnol. Oceanogr.* **56**, 1866-1878 (2011). https://doi.org:10.4319/lo.2011.56.5.1866

9 Stukel, M. R., Ohman, M. D., Benitez-Nelson, C. R. & Landry, M. R. Contributions of mesozooplankton to vertical carbon export in a coastal upwelling system. *Mar. Ecol. Prog. Ser.* **491**, 47-65 (2013). https://doi.org:10.3354/meps10453

10 Landry, M. R., Ohman, M. D., Goericke, R., Stukel, M. R. & Tsyrklevich, K. Lagrangian studies of phytoplankton growth and grazing relationships in a coastal upwelling ecosystem off Southern California. *Prog. Oceanogr.* **83**, 208-216 (2009). https://doi.org:10.1016/j.pocean.2009.07.026

11 Conover, R. J. Assimilation of organic matter by zooplankton. *Limnol. Oceanogr.* **11**, 338-345 (1966).

12 Moore, A. M. *et al.* The Regional Ocean Modeling System (ROMS) 4-dimensional variational data assimilation systems Part I - System overview and formulation. *Prog. Oceanogr.* **91**, 34-49 (2011). https://doi.org:10.1016/j.pocean.2011.05.004

13 Miller, A. J., Song, H. & Subramanian, A. C. The physical oceanographic environment during the CCE-LTER Years: Changes in climate and concepts. *Deep-Sea Res. II* **112**, 6-17 (2015). https://doi.org:10.1016/j.dsr2.2014.01.003

14 Song, H. *et al.* Application of a data-assimilation model to variability of Pacific sardine spawning and survivor habitats with ENSO in the California Current System. *J. Geophys. Res. Oceans* **117**, C03009 (2012). https://doi.org:10.1029/2011jc007302

15 North, E. W., Hood, R. R., Chao, S. Y. & Sanford, L. P. Using a random displacement model to simulate turbulent particle motion in a baroclinic frontal zone: A new implementation scheme and model performance tests. *J. Mar. Sys.* **60**, 365-380 (2006). https://doi.org:10.1016/j.jmarsys.2005.08.003

16 Boyd, P. W., Claustre, H., Levy, M., Siegel, D. A. & Weber, T. Multi-faceted particle pumps drive carbon sequestration in the ocean. *Nature* **568**, 327-335 (2019). https://doi.org:10.1038/s41586-019-1098-2
