# Supplementary material for: Carbon sequestration by multiple biological pump pathways in a coastal upwelling biome
Source: Nat Commun. 2023 Apr 11;14:2024. doi: 10.1038/s41467-023-37771-8 (PMC10090055; doi:10.1038/s41467-023-37771-8)
Supplement: Supplementary file 3 — Description of Additional Supplementary Files [file 41467_2023_37771_MOESM3_ESM.pdf]

Description of Additional Supplementary Files

File Name: Supplementary Data 1
Description: Sinking particle flux measured using surface-tethered drifting sediment traps. Columns refer to cruise name, the number of the Lagrangian Cycle on that cruise, sinking organic carbon flux (mmol C m-2 d -1), and the standard error of replicate particle interceptor tubes (typically n = 3).

File Name: Supplementary Data 2
Description: Particulate organic carbon subduction rates (mmol C m-2 d -1) as a function of depth (m). First column is depth. The other columns show subduction rates for individual Lagrangian experiments which are labeled as “CruiseName-LagrangianExperiment#”.

File Name: Supplementary Data 3
Description: Carbon dioxide sequestration time (years) as a function remineralization depth (m). Columns are depth, mean sequestration time, and the standard deviation of sequestration time as derived from variability between simulated molecules released during different years.
