# Supplementary material for: Carbon sequestration by multiple biological pump pathways in a coastal upwelling biome
Source: Nat Commun. 2023 Apr 11;14:2024. doi: 10.1038/s41467-023-37771-8 (PMC10090055; doi:10.1038/s41467-023-37771-8)
Supplement: Supplementary file 7 — Reporting Summary [file 41467_2023_37771_MOESM7_ESM.pdf]

nature portfolio

Corresponding author(s): Michael R Stukel

Last updated by author(s): 3/27/2023

Reporting Summary

Nature Portfolio wishes to improve the reproducibility of the work that we publish. This form provides structure for consistency and transparency in reporting. For further information on Nature Portfolio policies, see our Editorial Policies and the Editorial Policy Checklist.

Statistics

For all statistical analyses, confirm that the following items are present in the figure legend, table legend, main text, or Methods section.

n/a	Confirmed	
☐	☒	The exact sample size (*n*) for each experimental group/condition, given as a discrete number and unit of measurement
☐	☒	A statement on whether measurements were taken from distinct samples or whether the same sample was measured repeatedly
☐	☐	The statistical test(s) used AND whether they are one- or two-sided<br>*Only common tests should be described solely by name; describe more complex techniques in the Methods section.*
☐	☒	A description of all covariates tested
☒	☐	A description of any assumptions or corrections, such as tests of normality and adjustment for multiple comparisons
☐	☒	A full description of the statistical parameters including central tendency (e.g. means) or other basic estimates (e.g. regression coefficient) AND variation (e.g. standard deviation) or associated estimates of uncertainty (e.g. confidence intervals)
☒	☐	For null hypothesis testing, the test statistic (e.g. *F*, *t*, *r*) with confidence intervals, effect sizes, degrees of freedom and *P* value noted<br>*Give P values as exact values whenever suitable.*
☒	☐	For Bayesian analysis, information on the choice of priors and Markov chain Monte Carlo settings
☒	☐	For hierarchical and complex designs, identification of the appropriate level for tests and full reporting of outcomes
☒	☐	Estimates of effect sizes (e.g. Cohen's *d*, Pearson's *r*), indicating how they were calculated

Our web collection on statistics for biologists contains articles on many of the points above.

Software and code

Policy information about availability of computer code

Data collection	No software was used for data collection.
Data analysis	The MITgcm model (https://mitgcm.readthedocs.io/en/latest/#) v1.17<br>Larval TRANSport Lagrangian model v.1 (http://northweb.hpl.umces.edu/LTRANS.htm)

For manuscripts utilizing custom algorithms or software that are central to the research but not yet described in published literature, software must be made available to editors and reviewers. We strongly encourage code deposition in a community repository (e.g. GitHub). See the Nature Portfolio guidelines for submitting code & software for further information.

Data

Policy information about availability of data

All manuscripts must include a data availability statement. This statement should provide the following information, where applicable:

- Accession codes, unique identifiers, or web links for publicly available datasets
- A description of any restrictions on data availability
- For clinical datasets or third party data, please ensure that the statement adheres to our policy

In situ data used in this study and CCE monthly state estimates used for the subduction model are available through the CCE LTER Datazoo repository (https://oceaninformatics.ucsd.edu/datazoo/catalogs/ccelter/datasets), the Zooscan Database (https://oceaninformatics.ucsd.edu/zooplankton/zooscandb), and/or the Environmental Data Initiative (doi:10.6073/pasta/de679918c44266dcebbc5f85a37dcd36, doi:10.6073/pasta/d19f13b361177f7d10135d48498fa7c0, doi:10.6073/

pasta/4c90b0a9fca143c5203ba02027030555, doi:10.6073/pasta/3c607138a88218846cae4d6f201942f6, doi:10.6073/pasta/0a0d884667f55ffe551e33dcf7ebe535). HYCOM data used can be obtained from the Naval Research Laboratory (https://www.hycom.org/dataserver/gofs-3pt1/reanalysis). Data is also available in supplementary tables S1 – S3, Supplementary Data #1 – 3, and in a source data file that can be used to recreate figures.

Human research participants

Policy information about studies involving human research participants and Sex and Gender in Research.

Reporting on sex and gender	n/a
Population characteristics	n/a
Recruitment	n/a
Ethics oversight	n/a

Note that full information on the approval of the study protocol must also be provided in the manuscript.

Field-specific reporting

Please select the one below that is the best fit for your research. If you are not sure, read the appropriate sections before making your selection.

☐ Life sciences ☐ Behavioural & social sciences ☒ Ecological, evolutionary & environmental sciences

For a reference copy of the document with all sections, see nature.com/documents/nr-reporting-summary-flat.pdf

Ecological, evolutionary & environmental sciences study design

All studies must disclose on these points even when the disclosure is negative.

Study description	Data was collected during Lagrangian experiments conducted in different locations along the productivity gradient within the California Current Ecosystem on multiple cruises of the CCE LTER Program.
Research sample	The research sample included carbon flux measurements that were made during CCE LTER cruises.
Sampling strategy	Sampling was done as possible (i.e., we sampled on 11 different month-long cruises of the CCE LTER Program). We cannot sample when there is no ship available. Sample size was not pre-determined, but rather we collected as much data as possible. Monte Carlo Methods were used to determine resulting confidence limits based on sampling density.
Data collection	Data was collected by the multiple participants of the CCE LTER Program
Timing and spatial scale	The first samples were collected in May 2006. The last samples were collected in August 2021. Samples were only collected while we were out at sea (11 ~month long cruises, scattered throughout that time span)
Data exclusions	No data were excluded.
Reproducibility	Replicate measurements were taken (three independent tubes per sinking carbon flux measurements). Reproducibility of experimental design is not relevant, per se, because if you go to the same spot in the ocean on a different day conditions will have changed and hence measurements will be different.
Randomization	N/A
Blinding	N/A

Did the study involve field work? ☒ Yes ☐ No

Field work, collection and transport

Field conditions	The study was conducted on CCE LTER DataZoo cruises. Relevant meteorological conditions can be found on the CCE LTER DataZoo website.
Location	32 - 37N, 118 - 131W

Access & import/export	Sampling was done in the open ocean. No permits were required.
Disturbance	N/A

Reporting for specific materials, systems and methods

We require information from authors about some types of materials, experimental systems and methods used in many studies. Here, indicate whether each material, system or method listed is relevant to your study. If you are not sure if a list item applies to your research, read the appropriate section before selecting a response.

Materials & experimental systems

n/a	Involved in the study
☒	☐ Antibodies
☒	☐ Eukaryotic cell lines
☒	☐ Palaeontology and archaeology
☒	☐ Animals and other organisms
☒	☐ Clinical data
☒	☐ Dual use research of concern

Methods

n/a	Involved in the study
☒	☐ ChIP-seq
☒	☐ Flow cytometry
☒	☐ MRI-based neuroimaging
